# Radiosensitization by enzalutamide for human prostate cancer is mediated through the DNA damage repair pathway

**DOI:** 10.1371/journal.pone.0214670

**Published:** 2019-04-01

**Authors:** Konjeti R. Sekhar, Jian Wang, Michael L. Freeman, Austin N. Kirschner

**Affiliations:** Department of Radiation Oncology, Vanderbilt University Medical Center, Nashville, Tennessee, United States of America; Northwestern University, UNITED STATES

## Abstract

Radiation therapy is often combined with androgen deprivation therapy in the treatment of aggressive localized prostate cancer. However, castration-resistant disease may not respond to testosterone deprivation approaches. Enzalutamide is a second-generation anti-androgen with high affinity and activity that is used for the treatment of metastatic disease. Although radiosensitization mechanisms are known to be mediated through androgen receptor activity, this project aims to uncover the detailed DNA damage repair factors influenced by enzalutamide using multiple models of androgen-sensitive (LNCaP) and castration-resistant human prostate cancer (22Rv1 and DU145). Enzalutamide is able to radiosensitize both androgen-dependent and androgen-independent human prostate cancer models in cell culture and xenografts in mice, as well as a treatment-resistant patient-derived xenograft. The enzalutamide-mediated mechanism of radiosensitization includes delay of DNA repair through temporal prolongation of the repair factor complexes and halting the cell cycle, which results in decreased colony survival. Altogether, these findings support the use of enzalutamide concurrently with radiotherapy to enhance the treatment efficacy for prostate cancer.

## Introduction

Radiation therapy is a standard treatment for localized prostate cancer and is commonly used in combination with androgen deprivation therapy (ADT) to enhance disease control rates [1]. However, local control is not achieved in nearly half of these cases, allowing the development of metastatic disease and nearly 30,000 deaths per year in the USA.[2] Thus, there is an urgent need for novel strategies that selectively sensitize prostate tumors and counteract resistance to radiation treatment.

ADT, also known as hormone therapy, for prostate cancer acts as a radiation sensitizer, enhancing the efficacy of radiation treatment. Several radiosensitization mechanisms for ADT have been implicated, most prominently the influence of androgen receptor (AR) activity on cell cycle checkpoint inhibition [3] and DNA damage repair pathway factors, which include DNA-PKcs [4,5] and transcriptional changes in expression of DNA repair genes [6]. The DNA repair pathway genes have been implicated in recurrence and resistance to ADT and other treatments in patients and preclinical models of prostate cancer [7,8].

However, the detailed factors and mechanism of radiation sensitization has not yet been well-established for second-generation anti-androgen enzalutamide (ENZ)[9]. ENZ is thought to function by multiple mechanisms that interfere with the activity of the androgen receptor, including acting as an androgen antagonist, inhibiting nuclear import of androgen receptor (AR), and blocking the binding of AR to DNA [9]. Although some AR-mediated mechanisms by which ENZ influences sensitivity to radiation treatment are known, it remains unknown as to whether ENZ can radiosensitize in AR-independent (castration-resistant) prostate cancer and what factors may be involved in this process.

Second-generation ADT agents, such as ENZ and abiraterone, are approved therapies for metastatic prostate cancer. These agents may be used for hormone-sensitive and castration-resistant metastatic disease. Recently, second-generation ADT agents are being investigated as first-line therapeutics in combination with local therapies, such as radiation therapy. Thus, it is important to understand how second-generation ADT agents cause increased sensitivity to radiation therapy and may contribute to improving disease control rates.

The overall aim of this work is to demonstrate the mechanisms by which ENZ acts as a radiation sensitizer to improve the efficacy of radiation therapy for the treatment of both androgen-dependent and androgen-independent human prostate cancer. This work includes cell lines in tissue culture as well as xenografts in mouse models, including a patient-derived tumor that has failed multiple prior lines of therapy. Through these models, key factors involved in the mechanism of radiation sensitivity are demonstrated to be influenced by ENZ therapy.

## Materials and methods

### Cell lines

Three human prostate cancer cell lines were used in this work: hormone-sensitive LNCaP and 22Rv1, and castration-resistant DU145 (all obtained from ATCC). Cells were maintained in RPMI 1640 medium (Gibco) supplemented with 10% FBS (Hyclone) and 1% penicillin-streptomycin (Gibco).

### Antibodies

Antibodies were used according to the manufacturer’s instructions. Reagents include phospho-γH2AX(S139) (05636, Millipore), DNA-PKcs (MS423P0, Fisher Scientific), phospho-ATM (sc-47739, Santa Cruz), Ku-86 (sc-9034, Santa Cruz), β-Actin (sc-47778, Santa Cruz and A2066, Sigma), 53BP1 (NB100-304, Novus Biologicals), p300 (554215, BD Biosciences), TATA Binding Protein (8515, Cell Signaling).

### Colony formation assays

These assays were performed according to published methods [10]. Cell survival following ENZ + XRT is corrected for ENZ-mediated toxicity.

### Tumor grafts

For cell line xenografts, 1 million cells were put into 0.2 mL medium containing 50–70% Matrigel (BD Biosciences) and injected subcutaneously into the mouse hindlimb. Castration-resistant human patient-derived xenograft (PDX), TM00298 (Jackson Laboratory), is from a 71 year-old man with prostate cancer treated with radiation therapy to the prostate, ADT, and docetaxel chemotherapy. After all these treatments, TM00298 is derived from the primary prostate cancer site by a transurethral resection of the prostate (TURP) with chips positive for poorly differentiated carcinoma. Serial passaging of PDX was performed per the supplier’s protocol, which includes sterile transfer from donor to recipient NSG mice with subcutaneous grafts approximately 3 mm in size. PDX grafts were implanted subcutaneously into the inferior flank region.

### Drug and radiation treatment

Treatments were given to animals with tumor grafts that were at least 500 mm^3^ as measured by digital calipers using the modified ellipsoidal formula: 0.5 x length x width^2^ [11]. Enzalutamide (ENZ) drug was obtained directly from the manufacturer (Astellas-Medivation) and used in accordance with their recommendations and at doses previously shown relevant for hormone-sensitive and castration-resistant prostate cancer models [12,13]. For cell culture, ENZ was dissolved in dimethyl sulfoxide (DMSO) and assays were performed with a final concentration of 0.1% DMSO in cell growth medium. For mouse models, ENZ was dissolved in a vehicle solution of 0.5% methylcellulose A4M premium (Methocel, Sigma) in sterile 18 MΩ purified water, and it was administered by oral gavage at a concentration of 25 mg/kg/day. Radiation treatment (XRT) was given by orthovoltage X-ray source (Pantak, 300 kVp/10 mA) to the hindlimb or inferior flank location of the tumor, while shielding the majority of the animal body. The radiation dose of 2 Gy was given on consecutive weekdays 1–2 hours after ENZ/vehicle administration. Radiation dose was confirmed by film dosimetry using GAFChromic EBT-XD film (Ashland, Bridgewater, NJ), which has a broad dose range and minimal energy-dependence, calibrated to a clinical linear accelerator.

### Animals

All research involving vertebrate animals was performed in strict accordance with protocols M/14/182 and M1700134 approved by Vanderbilt’s Institutional Animal Care and Use Committee (IACUC). All procedures were conducted according to applicable national guidelines, including appropriate analgesics and anesthesia to ameliorate and minimize animal suffering. Tumor grafts from human cell lines were grown in nude mice (Foxn1^nu/nu^, Jackson Laboratory). Mice were monitored at least twice per week and body condition score was used to assess their well-being [14]. The maximum tumor size for a 25 g mouse was a diameter of 2 cm (4.2 cm^2^). Humane endpoints included when tumors reached the maximum size or became ulcerated, tumor affected gait, posture, ability to eat/drink/urinate/defecate, mice experienced lethargy, change in ambulation, diarrhea, increased respiratory effort, or neurological signs, or when mice lost more than 20% of pre-treatment body weight (body condition score 1 or 2 with decreased activity). Mice were sacrificed under anesthesia (inhaled isoflurane) using cervical dislocation.

### Immunofluorescence

LNCaP cells were plated on coverslips and treated with either DMSO or ENZ (10 μM) for 24–48 hrs. Cells were irradiated with 0, 1, or 2 Gy, and then at specific time-points were fixed with formaldehyde. The cells were permeabilized with Triton X-100 and blocked with 5% BSA. Antibodies were applied to detect relevant proteins: γH2AX-Alexa647 (BD Farmingen, Cat#560447, 1:1000 dilution), and 53BP1 (NB100-304, Novus Biologicals) with secondary anti-rabbit-IgG-Alexa568 (Life Technologies, Cat # A11011, 1:1000 dilution). DAPI was used as a counterstain in the mounting medium on the glass slide. Slides kept in the dark were cured overnight at room temperature and stored at 4°C. Quantification of immunfluorescent foci was performed by quantifying the average number of foci per cell (4 fields per slide) and subtracting the background foci quantified from the untreated (DMSO control) unirradiated sample.

### Tissue analysis

Whole slide imaging and quantification of immunostaining were performed in the Digital Histology Shared Resource at VUMC (www.mc.vanderbilt.edu/dhsr). Immunostained tissue slides were imaged on a Leica SCN400 Slide Scanner (Leica Biosystems) at 20X magnification to a resolution of 0.5 μm/pixel. Individual cells were identified utilizing standard Ariol analysis scripts (Leica). Upper and lower thresholds for color, saturation, intensity, size, roundness, and axis length were set for both blue Hematoxylin staining of nuclei and for brown DAB reaction products. Thus, brown (DAB) positive cells can be distinguished from blue (Hematoxylin only) negative cells. Uniform thresholds were applied for degree of DAB staining and cell size to systematically determine positive cells. The percentage of positive cells was determined as the positive stained cells divided by the total cells counted.

### Immunoblots

Whole cell lysate or nuclear lysate was extracted from cultures using RIPA buffer (10 mM Tris, pH 8.0, 150 mM NaCl, 5 mM EDTA, 0.1% SDS, 0.1% Triton X-100, 1% deoxycholic acid) or nuclear extraction buffer (50 mM Tris, pH 7.4, 50 mM NaCl, 10 mM EDTA, 0.5% NP-40), both containing protease inhibitors (Complete Mini, Sigma Aldrich) and phosphatase inhibitors (PhosStop, Sigma Aldrich). Protein concentration was determined by light absorption method (DC Protein Assay Kit, Bio-Rad). SDS-PAGE was performed using 4–12% precast gradient gel (Nupage, Life Technologies) followed by transfer to 0.45 μm PVDF membrane (Hybond, GE Healthcare Amersham). Immunoblotting was performed in TBS-T with up to 10% dry nonfat milk blocking reagent.

## Results

### DNA-damage response

One of the key mechanisms of radiosensitization involves alterations in the DNA damage-repair pathway. Therefore we first tested the hypothesis that ENZ impaired repair of DNA damage. The early-responding elements of DNA damage-repair pathway were studied to determine whether ENZ alters the initial factors involved in recognition and signaling of DNA damage.

**Fig 1:**
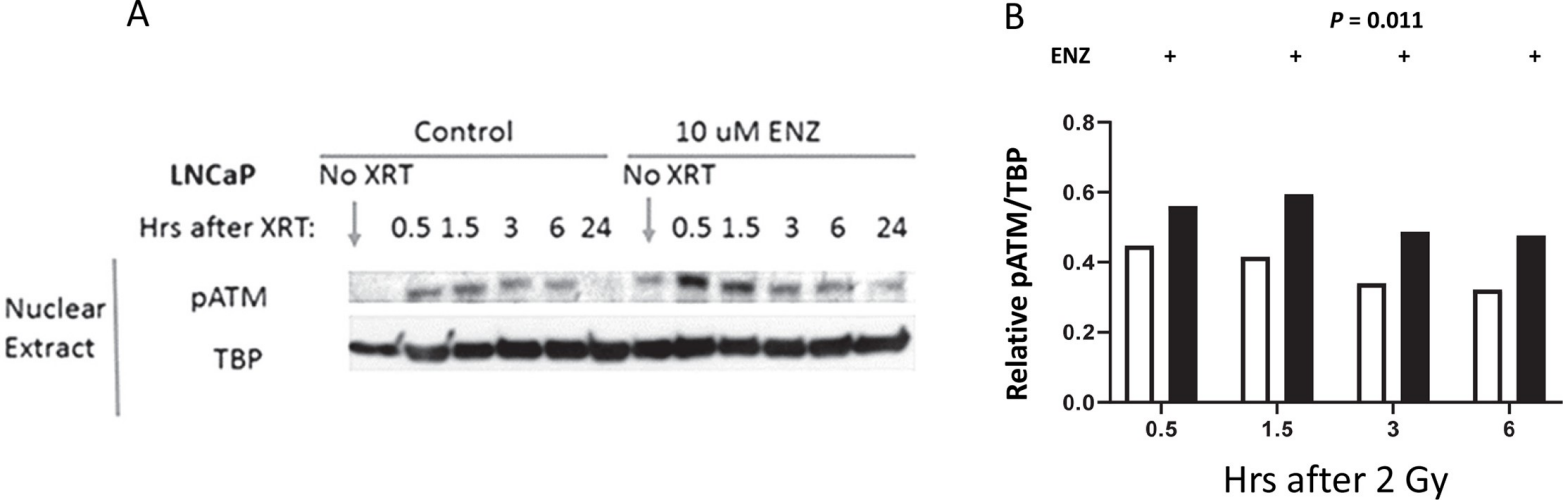
LNCaP cells were exposed to 10 μM ENZ for 24 hrs prior to, during irradiation (2 Gy) and for up to 24 hrs after irradiation. At the indicated times cells were processed for immunoblotting. A) LNCaP nuclear lysates immunoblotted for pS1981 ATM and TBP (TATA-binding protein). No XRT represents sham treatment. B) Ratio of pS1981 ATM immunoreactive protein corrected for TBP immunoreactive protein.

Autophosphorylation of ATM at S1981 represents one of the earliest markers of DNA double strand breaks [15]. Immunoblotting (Fig 1A) was used to quantify expression of pS1981 ATM in irradiated (2 Gy) human prostate cancer LNCaP cells exposed to solvent control or ENZ (Fig 1B). The data indicate that exposure to 10 μM ENZ for 24 hrs prior to, during, and for up to 6 hrs after irradiation resulted in a 2 to 3 fold increase in pS1981 ATM compared to cells treated with solvent control (DMSO, *P* = 0.0087). Twenty-four hrs after irradiation pS1981 ATM levels returned to control levels (Fig 1). The degree of S1981 ATM phosphorylation has been shown to be proportional to the number of DNA double strand breaks [16]. In addition, unirradiated cells demonstrate a relatively smaller increase in ATM phosphorylation due to ENZ treatment, which has been previously reported [17]. Thus, one possibility was that the ENZ exposure impaired repair of DNA double strand breaks present in irradiated cells.

**Fig 2:**
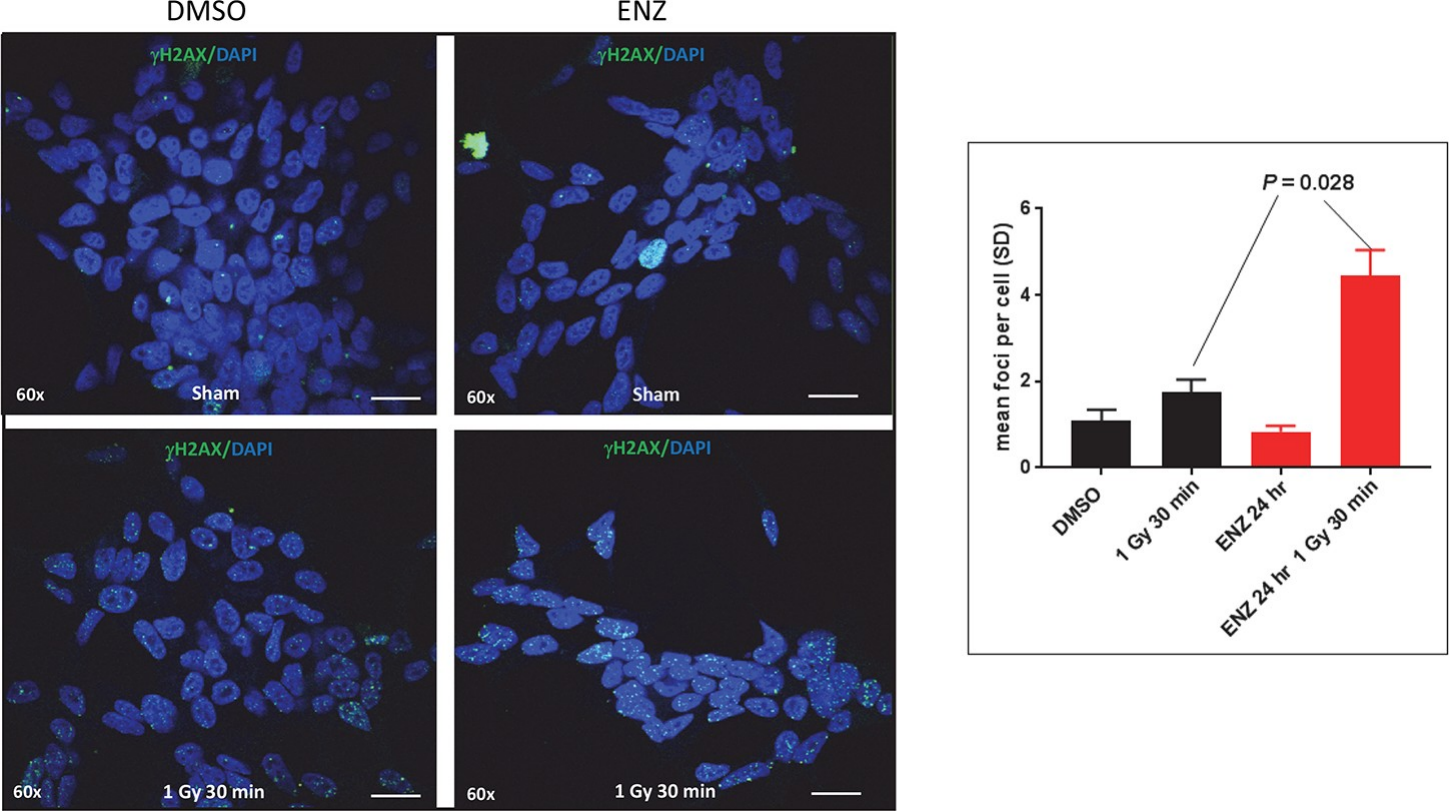
LNCaP cells in culture were treated for 24 hrs with DMSO or ENZ, then irradiated with 0 or 1 Gy. One half hour after irradiation cells were fixed and immunostained for γH2AX foci formation. Immunofluorescent images were obtained by confocal microscopy. White bar = 20 microns. A) Representative confocal images. B) Analysis of the mean number of foci per cell (± SD).

**Fig 3:**
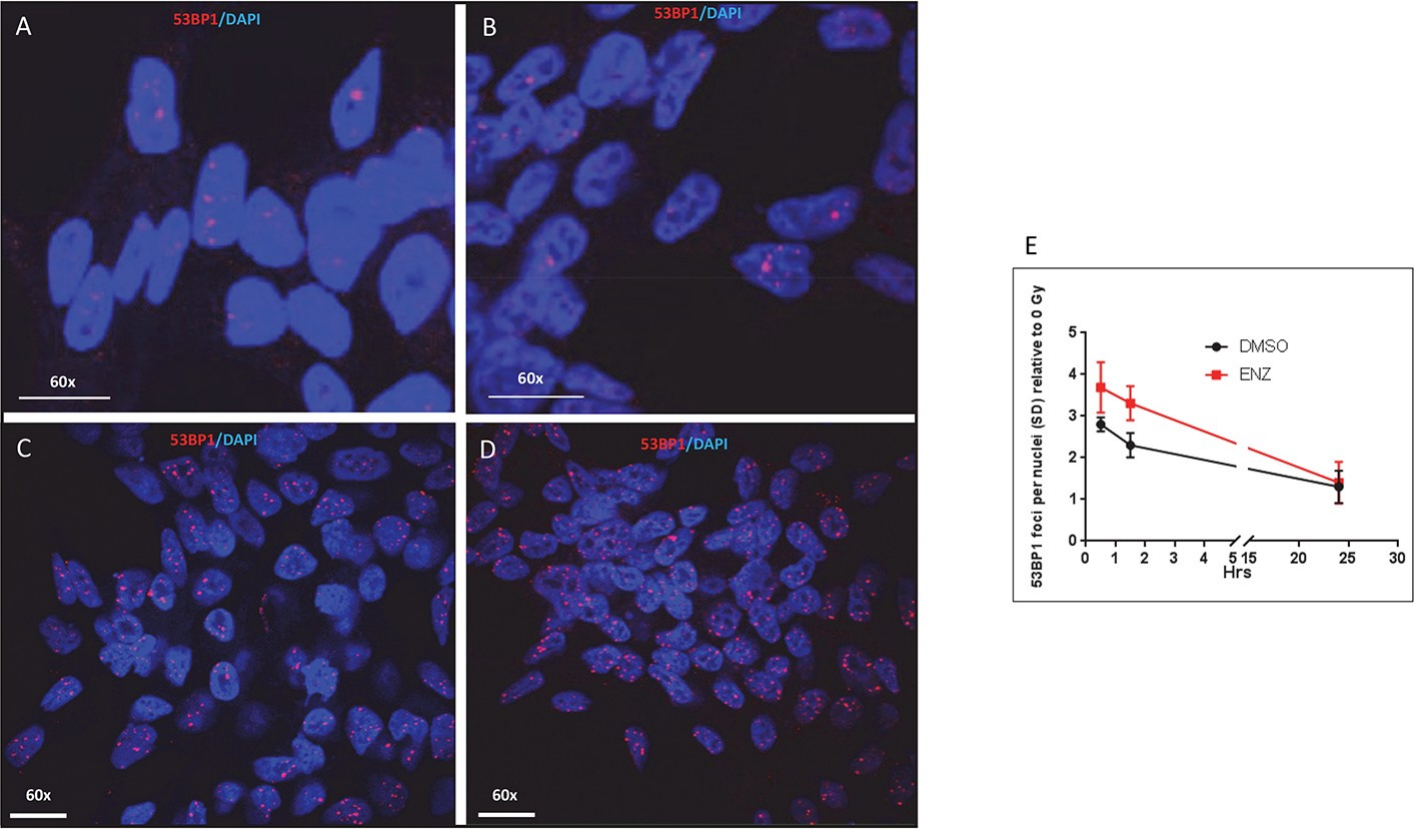
LNCaP cells in culture were treated for 24 hrs with DMSO or ENZ, then irradiated with 0 or 1 Gy. Cell were fixed and immunostained for 53BP1 0.5, 1.5 and 24 hrs after irradiation. Immunofluorescent images were obtained by confocal microscopy. White bar = 20 microns. A) DMSO alone, B) ENZ alone, C) 1 Gy, D) ENZ + 1 Gy. White bar = 20 microns.

Activation of the phosphatidylinositol kinase-like kinase ATM results in rapid phosphorylation of Ser139 on the histone variant H2AX [18]. Thus, this phosphorylation event can be used as a surrogate to quantify radiation-induced DNA double strand breaks [19]. LNCaP cells were treated with DMSO or ENZ (10 μM) for 48 hours and then administered 0 or 2 Gy. Cells were fixed and immunostained for γH2AX foci 30 min after irradiation (Fig 2). An ENZ exposure significantly increased the number of radiation-induced foci (a 2.5 fold increase, *P* = 0.028, N = 475 nuclei). Immediately following phosphorylation of the histone H2AX MDC1 is recruited to and co-localizes with γH2AX [20]. MDC1 co-localization then promotes 53BP1 foci formation. We used 53BP1 foci formation and resolution as a surrogate for monitoring DNA double strand break repair. LNCap cells were treated with DMSO or ENZ (10 μM) for 48 hours and then administered 0 or 2 Gy. 53BP1 foci formation was monitored 0.5, 1.5 and 24 hrs after irradiation. At 0.5 and at 1.5 hrs there were significantly more 53BP1 foci in irradiated, ENZ treated and irradiated cells compared to DMSO treated and irradiated cells (*P* = 0.029 at 0.5 hrs, N = 1028 nuclei and *P* = 0.0073 at 1.5 hrs, N = 1028 nuclei, Fig 3E). The increase in pATM, γH2AX, and 53BP1 foci formation observed in irradiated ENZ-treated cells suggests the hypothesis that ENZ inhibits repair of radiation-induced DNA double strand breaks.

### Radiosensitization, in vitro

**Fig 4:**
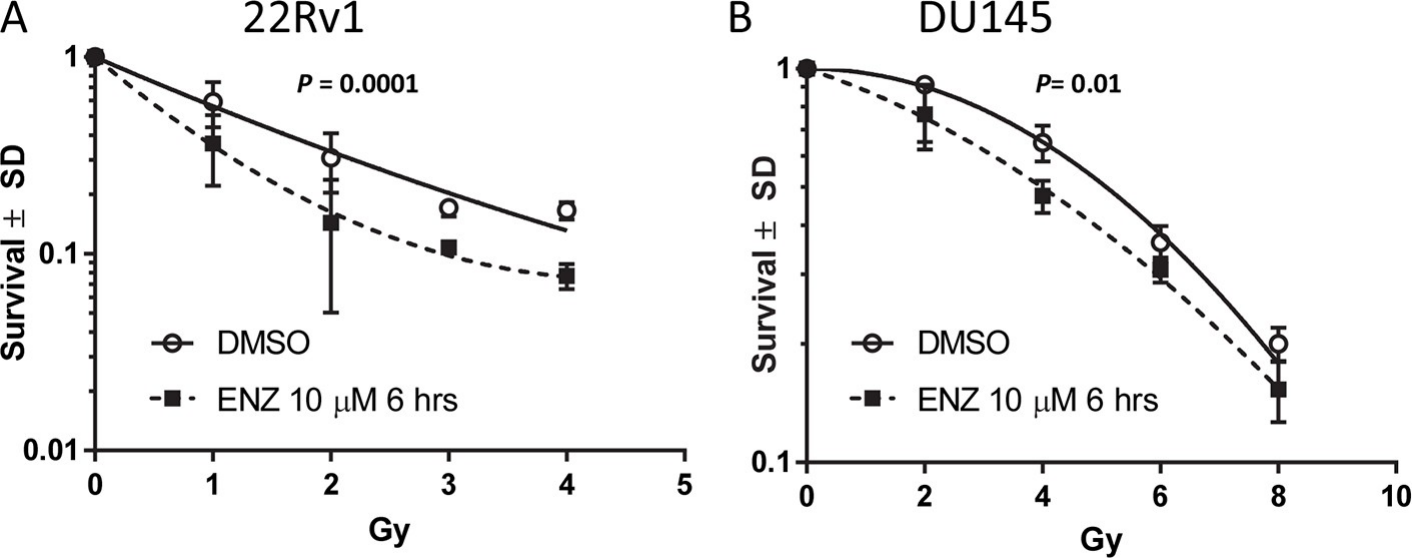
ENZ treatment radiosensitizes. A) 22Rv1 cells and B) DU145 cells were exposed to ENZ (10 μM) or DMSO 48 hrs and then administered the indicated doses. Six hours after irradiation the cells were washed extensively in fresh growth medium. Colony formation was quantified 14 days after irradiation. The data were fitted to the equation S = e^-αD-βD^2^.

Quantitative colony formation assays were performed to determine the impact of ENZ on radiosensitization of androgen-responsive 22Rv1 cells and androgen-independent DU145 cells that are not dependent on testosterone for growth [21]. Cells were exposed to 0 (DMSO) or 10 μM ENZ for 48 hrs, administered 0, 1, 2, 3 or 4 Gy, incubated at 37°C for 6 hrs, and then washed extensively. Cell survival was calculated from the resulting colony formation 14 days after irradiation. The data were fit to the equation S = e^-αD-βD^2^ (Fig 4). For 22Rv1 cells a single value for α and a single value for β could not adequately describe both the DMSO and the ENZ data points (Sum of Squares F test). The DMSO + irradiation data were best fitted with α = 0.59 and β = - 0.022, R^2^ = 0.88. For the ENZ + irradiation the best fit was α = 1.17 and β = -0.13, R^2^ = 0.90, *P* = 0.0001. A similar analysis was performed for the data obtained using DU145 cells. Again the analysis indicates that the DMSO + irradiation curve was significantly different from the ENZ + irradiation curve, *P* = 0.01. The data clearly indicate that ENZ treatment radiosensitizes prostate cancer cells, independent of androgen responsiveness.

### Radiosensitization, in vivo

**Fig 5:**
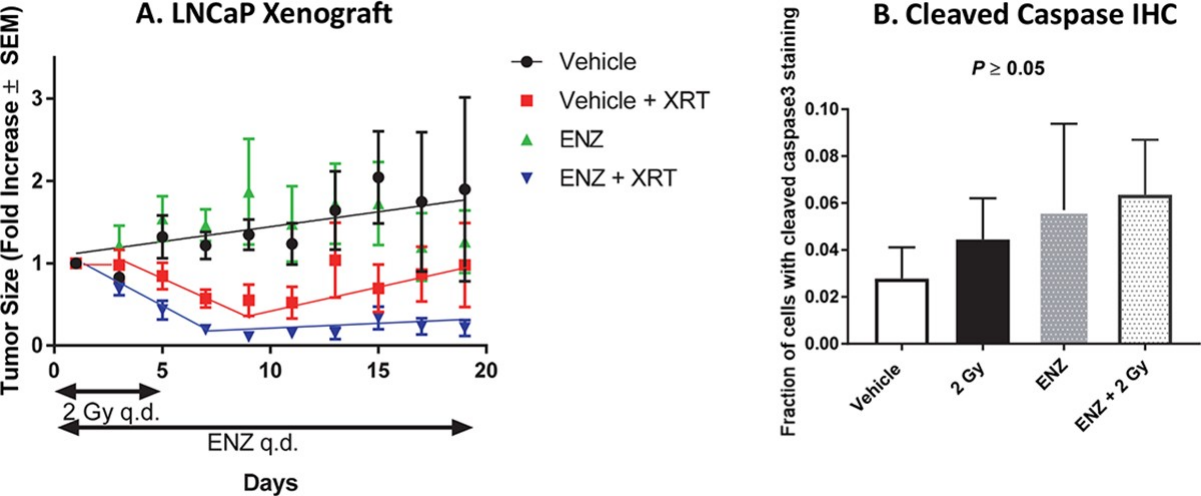
ENZ radiosensitizes xenograft tumors. A) LNCaP xenografts in nude mice were treated by vehicle (q.d. for 20 consecutive days), vehicle (q.d. for 20 consecutive days) + 2 Gy q.d. for 5 consecutive days, ENZ (q.d. for 20 consecutive days), or ENZ (q.d. for 20 consecutive days) + 2 Gy (q.d. for 5 consecutive days) (N = 5 per treatment group). B &C) LNCaP xenograft immunohistochemical staining, quantified by automated cell counting using standard Ariol analysis scripts (Leica) for detecting positive cells (DAB stain). B) Percent cells positive for cleaved caspase-3 (apoptosis marker). Total cells counted: 1,504,915, 1,914,981, 675,835, and 379,511 for vehicle, ENZ, 2 Gy, and ENZ+2Gy, respectively.

Human prostate cancer cell lines were engrafted in nude mice and used to examine the radiosensitization effects of ENZ *in vivo*. Mice with LNCaP xenografts (average tumor volume of 500 mm^3^) were treated with vehicle (q.d. for 20 days), vehicle (q.d. for 20 consecutive days)+ XRT (2 Gy/day for 5 consecutive days), ENZ (25 mg/kg q.d. for 20 consecutive days), or ENZ (q.d. for 20 consecutive days) + XRT (2 Gy/day for 5 consecutive days, Fig 5A). Treating tumor-bearing mice with ENZ alone did not result in a statistically significant impairment in tumor growth rate compared to vehicle alone (*P* = 0.31). Tumors treated with 2 Gy a day for 5 days (plus or minus ENZ treatment) experienced an initial decrease in tumor size compared to vehicle alone (*P* < 0.05). Whereas tumor progression resumed in tumor-bearing mice treated with radiation alone, irradiation plus ENZ treatment impaired progression (*P* < 0.0001).

LNCaP xenografts were harvested on day 20 and immunohistochemical (IHC) staining for cleaved caspase-3 was performed to determine the effect of treatment on apoptosis. Quantitative analysis of immunohistochemical staining indicated that irradiation (±ENZ treatment) resulted in a non-significant increase in apoptosis in LNCaP xenografts from mice treated with ENZ, 2 Gy, or ENZ+XRT compared to vehicle (Fig 5C, *P* > 0.05). This result is consistent with the knowledge that radiation-induced apoptosis in many solid tumors does not play a significant role in radiation-mediated effects.

**Fig 6:**
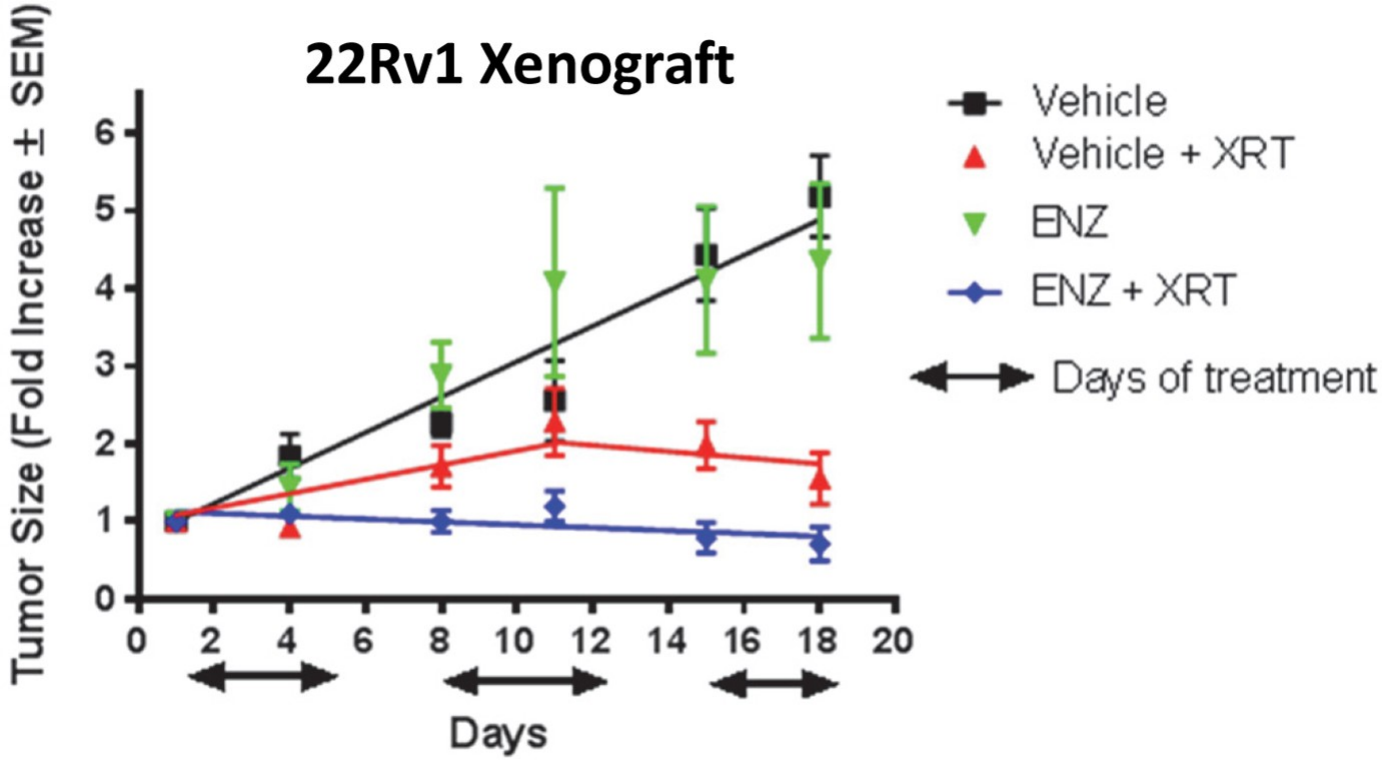
22Rv1 human prostate cancer xenografts in castrated nude mice were treated by vehicle, vehicle + XRT (2 Gy/day), ENZ (25 mg/kg/day), or ENZ + XRT, as described in the Results section (n = 5 per treatment group). Error bars are standard error of the mean (SEM).

A castration-resistant prostate cancer model was used next in which castrated nude mice were engrafted with human 22Rv1 cells. After xenografts reached an average volume of 500 mm^3^, mice were treated with by vehicle, vehicle + XRT (2 Gy/day), ENZ (25 mg/kg/day), or ENZ + XRT. Mice were treated for 5 consecutive days, allowed 2 days to recover, treated again for 5 consecutive days, allowed 2 days to recover, and then treated for 5 consecutive days (Fig 6). The rate of tumor progression in mice treated with ENZ alone was not statistically different from the rate of progression exhibited by tumors in mice treated with vehicle only (*P* = 0.89). Treatment with radiation alone significantly slowed tumor progression compared to vehicle alone or ENZ alone (*P* < 0.001). Importantly, addition of ENZ + XRT slowed tumor progression approximately 2-fold compared to Vehicle + XRT (*P* < 0.001, Fig 6). These data were interpreted to indicate that ENZ acts as a radiosensitizer for castration-resistant 22Rv1 human prostate cancer xenografts in a mouse model.

**Fig 7:**
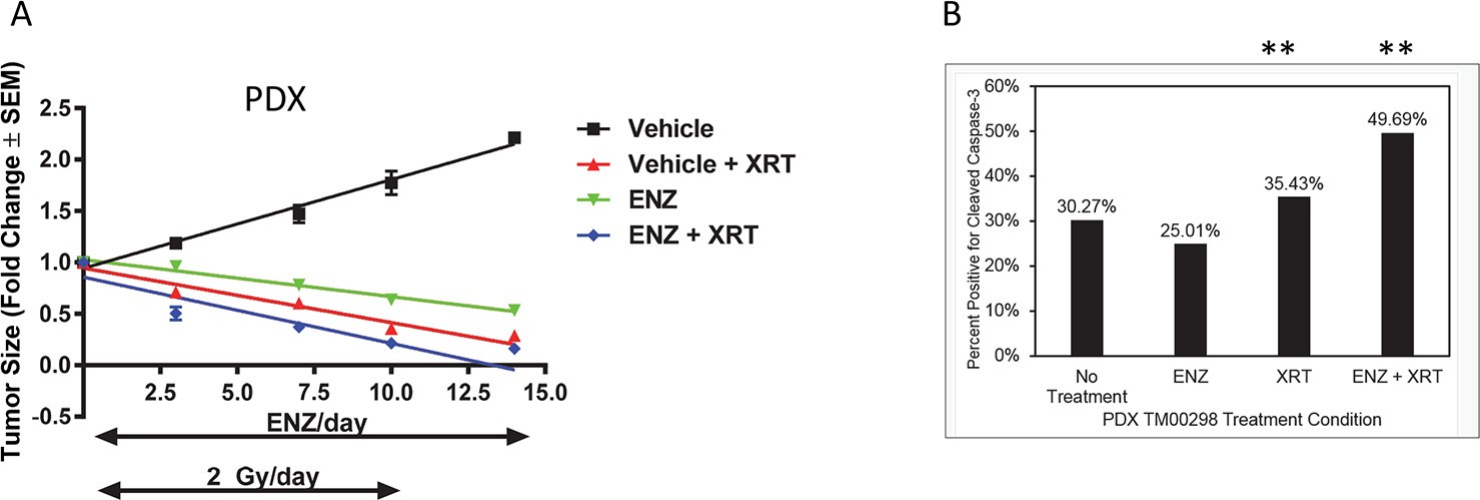
Response of a PDX model to ENZ and XRT. A) TM00298 human prostate cancer grafts were implanted into castrated NSG mice. Tumors were treated by vehicle, vehicle + XRT (2 Gy per day), ENZ (25 mg/kg per day), or ENZ + XRT (n = 5 per treatment group). ENZ is given once daily on weekdays. Treatment by XRT was 2 Gy x 10 weekday treatments (total 20 Gy). Error bars are standard error of the mean (SEM) and for some points the error bars are shorter than the height of the symbol. B) PDX TM00298 xenograft IHC staining, quantified by automated cell counting using standard Ariol analysis scripts (Leica) for detecting positive cells (DAB stain). Percent positive for cleaved caspase-3 staining relative to total cells quantified. Total cells counted 315,853, 522,978, 267,016, and 291,165 for vehicle, ENZ, XRT, and ENZ+XRT, respectively. ** *P* < 0.00001.

Lastly, a patient-derived xenograft (PDX) human prostate cancer model, TM00298, was studied in castrated NSG mice (Fig 7). After grafts were an average volume of 500 mm^3^, mice were treated with by vehicle, vehicle + XRT (2 Gy), ENZ (25 mg/kg), or ENZ + XRT. In this PDX model ENZ alone inhibited tumor proliferation compared to vehicle alone (*P* < 0.001). Administering 2 Gy a day for 10 consecutive weekdays (irradiation alone) was more effective in inhibiting tumor growth than the ENZ alone protocol (*P* < 0.001). Concurrent ENZ plus irradiation was the most effective (*P* = 0.0128 when compared to irradiation alone).

IHC analysis of the harvested PDX tumor tissue was undertaken to study changes related to the tumor volume changes observed. Cleaved caspase-3 was stained in the tumor as a marker for cells undergoing apoptosis. There was a statistically signficant increase in cleaved caspase-3 in the tumor grafts from mice treated with either XRT alone or ENZ+XRT compared to untreated mice (*P* < 0.00001). This suggests that part of the tumor growth inhibition mechanism involves an increase in apoptosis due to the radiosensitivity induced by ENZ+XRT treatment.

## Discussion

The prostate cancer cell lines studied in this project are well-established models of human prostate cancer and include both hormone-sensitive and castration-resistant/androgen-independent models. These prostate cancer cell culture and xenograft models were used to determine if concurrent ENZ and XRT resulted in radiosensitization. The treatment approaches were modeled to be clinically relevant, with adequate time for pre-treatment castration to result in androgen deprivation, oral administration route for ENZ, long duration of treatment (2–3 weeks), and daily treatments given on weekdays at clinically relevant radiation doses.

The studies described herein demonstrate that ENZ treatment functions to radiosensitize prostate cancer, a consequence of ENZ-mediated inhibition of DNA damage repair. Combining ENZ with irradiation increased the radiosensitivity of the AR-dependent cell line 22Rv1 and the AR-independent cell line DU145. Consistent with the cell culture models, we found that radiation-induced tumor growth delay was enhanced by concurrent ENZ treatment of LNCaP and 22Rv1 xenografts, as well as a TM00298 PDX tumor. Importantly, radiosensitization is observed at the clinically relevant dose of 2 Gy per fraction. The treatment effects on the DNA damage repair pathway appear to manifest as increased apoptosis, although it was statistically non-significant, possibly due to a small sample size. This may have implications for clinically relevant outcomes, since cleaved caspase-3 is a known prognostic biomarker for patients with localized prostate cancer [22]. This factor could be prospectively analyzed in clinical trials combining radiotherapy with enzalutamide.

Androgen receptor activity has been shown to be critical for repair of radiation-induced potentially lethal DNA double strand breaks [4,6]. Combining the second-generation AR inhibitor ENZ with XRT was found to impair repair of double strand breaks, evidenced by elevation of pATM, increased γH2AX foci, and increased 53BP1 foci, which are factors involved in the repair process. However, ENZ-mediated radiosensitization was observed in low AR-expressing prostate cancer cell line DU145. Although DU145 cells are not dependent on androgen for growth-related effects, they do express AR at a low level with RNA editing [23,24]. The completely AR-negative PC-3 prostate cancer cell line does not exhibit changes in proliferation, apoptosis, or other endpoints at ENZ doses of 10 μM or up to 40 μM in combination with radiotherapy [25]. Altogether, this suggests that ENZ may radiosensitize DU145 cells through the AR-pathway, which presumably still influences the repair of DNA double strand breaks, likely mediated through direct AR targeting of DNA repair genes [4,6]. Although the clonogenic assay supports radiosensization by ENZ for DU145 cells, a limitation of this work is that gene expression analysis was not performed to support this mechanism. Alternative explanations for the radiosensitization may include non-AR-mediated mechanism or less likely due to off-target effects from ENZ at 10 μM dose in cell culture.

PDX models have the benefits of including tumor heterogeneity, maintaining original histology and genetic expression profiles, and lacking changes that occur from cell line propagation. Although the human prostate cancer cell lines in this project are standard models for prostate cancer, they individually represent a limited aspect of human disease, such as a single metastatic phenotype. The PDX used in this project, TM00298, is a very unique tumor sample that has survived despite treatment with prostate radiotherapy, ADT, and docetaxel chemotherapy, as well as being derived from the primary prostate site, not a metastatic site. Thus, it represents a highly treatment-resistant human prostate tumor and is an excellent model in which to explore the effect of a second-generation ADT agent, such as ENZ. Through the observed tumor growth delay and increase in apoptosis, ENZ-mediated radiosensitivity is demonstrated in the PDX model, which supports the findings of the impact of ENZ and XRT on human prostate cancer cell lines.

In July 2018, the FDA approved enzalutamide for patients with non-metastatic CRPC based on the randomized clinical trial PROSPER (NCT020032924), in addition to its prior 2012 approval for metastatic CRPC progressing after docetaxel chemotherapy [26,27]. Ongoing clinical trials are investigating the combination of ENZ with radiotherapy in patients with intermediate risk localized prostate cancer (NCT02028988, NCT02023463, ENZART NCT03196388), high-risk localized prostate cancer (NCT02064582, ENZARAD NCT02446444, and NCT02508636), and in patients post-prostatectomy receiving salvage radiation therapy for recurrent prostate cancer (STREAM NCT02057939 and NCT02203695). This project provides key mechanistic data to support the superior tumor-killing effect of concurrent ENZ with radiation therapy for the localized treatment of both hormone-sensitive and castration-resistant prostate cancer.

## Conclusions

ENZ causes radiosensitization in human prostate cancer that is mediated through decreased DNA repair, which is manifested by increased expression and temporal persistence of DNA damage repair factors. Through testing in AR-dependent and AR-independent prostate cancer cell lines as well as a treatment-resistant patient-derived xenograft, this work provides fundamental mechanistic support for the use of ENZ as a radiosensitizer to be given simultaneously with XRT in the treatment of prostate cancer.

## Supporting information

S1 Appendix Raw data and details of the figures/graphs are included in the S1 Appendix spreadsheet file attached to this manuscript. (XLSX)
